# An autocrine inflammatory forward-feedback loop after chemotherapy withdrawal facilitates the repopulation of drug-resistant breast cancer cells

**DOI:** 10.1038/cddis.2017.319

**Published:** 2017-07-13

**Authors:** Deyong Jia, Li Li, Sulaiman Andrew, David Allan, Xuguang Li, Jonathan Lee, Guang Ji, Zemin Yao, Suresh Gadde, Danial Figeys, Lisheng Wang

**Affiliations:** 1 Department of Biochemistry, Microbiology and Immunology, Faculty of Medicine, University of Ottawa, Ottawa, Ontario, Canada; 2 China-Canada Centre of Research for Digestive Diseases, Ottawa, Ontario, Canada; 3 Institute of Digestive Diseases, Longhua Hospital, Shanghai University of Traditional Chinese Medicine, Shanghai, China; 4 Regenerative Medicine Program, Ottawa Hospital Research Institute, Ottawa, Ontario, Canada; 5 Centre for Biologics Evaluation, Biologics and Genetic Therapies Directorate, Health Canada Sir Frederick G. Banting Research Centre, Ottawa, Ontario, Canada; 6 Ottawa Institute of Systems Biology, University of Ottawa, Ottawa, Ontario, Canada

## Abstract

Stromal cells, infiltrating immune cells, paracrine factors and extracellular matrix have been extensively studied in cancers. However, autocrine factors produced by tumor cells and communications between autocrine factors and intracellular signaling pathways in the development of drug resistance, cancer stem-like cells (CSCs) and tumorigenesis have not been well investigated, and the precise mechanism and tangible approaches remain elusive. Here we reveal a new mechanism by which cytokines produced by breast cancer cells after chemotherapy withdrawal activate both Wnt/*β*-catenin and NF-*κ*B pathways, which in turn further promote breast cancer cells to produce and secrete cytokines, forming an autocrine inflammatory forward-feedback loop to facilitate the enrichment of drug-resistant breast cancer cells and/or CSCs. Such an unexpected autocrine forward-feedback loop and CSC enrichment can be effectively blocked by inhibition of Wnt/*β*-catenin and NF-*κ*B signaling. It can also be diminished by IL8-neutralizing antibody or blockade of IL8 receptors CXCR1/2 with reparixin. Administration of reparixin after chemotherapy withdrawal effectively attenuates tumor masses in a human xenograft model and abolishes paclitaxel-enriched CSCs in the secondary transplantation. These results are partially supported by the latest clinical data set. Breast cancer patients treated with chemotherapeutic drugs exhibited poor survival rate (66.7 *vs* 282.8 months, *P*=0.00071) and shorter disease-free survival time if their tumor samples expressed high level of *IL8*, *CXCR1*, *CXCR2* genes and Wnt target genes. Taken together, this study provides new insights into the communication between autocrine niches and signaling pathways in the development of chemotherapy resistance and CSCs; it also offers a tangible approach in breast cancer treatment.

Breast cancer is a leading cause of death in women, with about 1.7 million new cases and more than half a million deaths in the world each year.^1^ Despite considerable advances, most chemotherapeutic regimens that are administered at intervals to avoid irreparable damage to vital host functions ultimately fail to control disease progression.^2^ This is largely due to the development of drug resistance and the recovery and repopulation of drug-resistant tumor cells between treatment cycles.^3, 4, 5^

Although acquired drug resistance is frequently due to the reactivation of signaling pathways suppressed during therapies, treatments that block one pathway are not durable and are less effective when treating cancer recurrence.^6, 7, 8, 9, 10^ In addition, drug-resistant cells and/or cancer stem-like cells (CSCs) capable of initiating new tumors *in vivo* have been considered as key cellular compartments in cancer recurrence.^11, 12^ The driving forces behind drug resistance and CSC development have been closely linked to pathways that mediate communication networks between tumor cells, inflammatory factors, and other microenvironment niches.^13, 14^

Stromal cells, infiltrating immune cells, paracrine factors and extracellular matrix components contribute to cancer microenvironments that have been extensively studied.^15^ However, autocrine factors produced by tumor cells and their communication with intracellular signaling pathways in drug resistance, CSC development and tumorigenesis after chemotherapy withdrawal have not been well investigated, and precise mechanistic insight remains lacking. Cytokines (such as IL6, IL8 and CCL2) and their signaling pathways have been demonstrated to have important roles in breast cancer initiation, migration, invasion and disease progression.^16, 17, 18^ However, it remains unclear whether breast cancer cells are capable of producing large amount of cytokines acting as autocrine factors to self-propel the development of drug resistance and CSCs after chemotherapy withdrawal.

In this study, we show that breast cancer cells increase production and secretion of IL6, IL8, CSF2 and CCL2 cytokines after withdrawal of chemotherapeutic drugs (paclitaxel, 5-fluorouracil or doxorubicin). These cytokines activate both NF-*κ*B and Wnt/*β*-catenin pathways, which in turn promote the production and secretion of more cytokines, forming an autocrine inflammatory forward-feedback loop to enrich drug-resistant cancer cells and CSCs. Accordingly, inhibition of NF-*κ*B and Wnt/*β*-catenin pathways, neutralization of IL8 or inhibition of IL8 receptor CXCR1/2 with reparixin significantly diminishes forward-feedback loop and CSC enrichment *in vitro*. Administration of reparixin after paclitaxel withdrawal not only effectively attenuates tumor masses in a human breast cancer xenograft model, but also significantly inhibits the enrichment of CSCs and the growth of paclitaxel-resistant breast cancer cells in secondary transplantation.

## Results

### The supernatants derived from breast cancer cells four days after drug withdrawal enhance CSC phenotypes and chemoresistance

**Figure 1:**
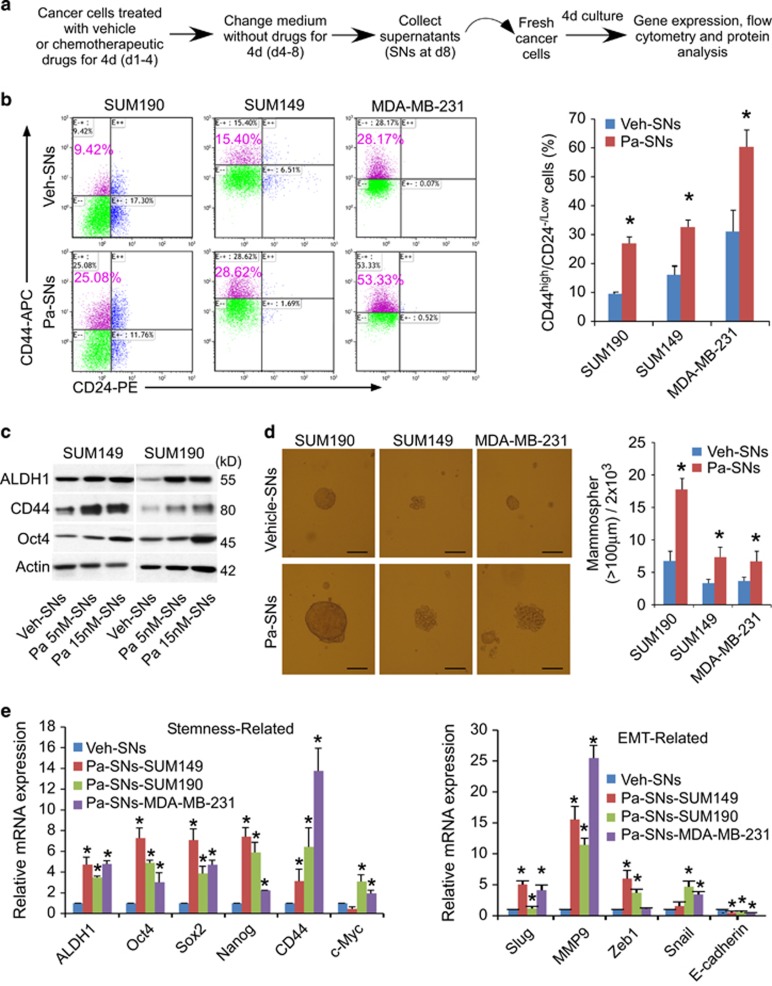
The supernatants derived from breast cancer cells four days after drug withdrawal enhance CSC phenotypes and chemoresistance. (**a**) Experimental design for the study of autocrine factors in supernatants derived from chemotherapy-treated breast cancer cells. SUM190, SUM149 and MDA-MB-231 cells were treated with 15 nM paclitaxel for 4 days. After medium change and wash with PBS, the cells were cultured in fresh medium in the absence of any drug for additional 4 days. At day 8, the cell-free and drug-free culture media (Supernatants) were collected and applied to the same line of fresh cells respectively for 4 days. (**b**) Flow cytometric analysis of the percentage of CD44^high^/CD24^-/low^ cells in SUM190, SUM149 and MDA-MB-231 cells after exposure to the supernatants for 4 days. The 7-AAD-negative live cells were pre-gated. Data represent means±S.D., *n*=3; * *P*<0.05. Veh-SNs: supernatants (SNs) derived from vehicle (Veh)-pretreated cells; Pa-SNs: supernatants derived from 15 nM paclitaxel (Pa)-pretreated cells. (**c**) Western blot analysis of stem cell-like markers (ALDH1, CD44 and OCT4) in SUM149 and SUM190 cells after exposure to the supernatants for 4 days. Supernatants (SNs) were collected as described in **a** and **b**. Pa 5 nM-SNs or Pa 15 nM-SNs: pretreated with 5 or 15 nM paclitaxel. *β*-actin was used as an internal loading control. (**d**) Representative images from mammosphere assays. SUM190, SUM149 and MDA-MB-231 breast cancer cells were treated for 4 days with vehicle (Veh)-derived or paclitaxel (Pa)-derived supernatants (SNs), and then reseeded in ultra-low attachment plate (2 × 10^3^/well, 6-well plate) and cultured for 8 days. Scale bar, 100 *μ*m. Data represent means±S.D., *n*=3; *P<0.05. (**e**)qPCR analysis of stemness-associated (*ALDH1*, *SOX2*, *OCT4*, *NANOG*, *CD44* and c-*MYC*), epithelial-to-mesenchymal transition (EMT)- (*SLUG*, *MMP9*, *ZEB1*, *SNAIL* and *E-CADHERIN*) signature genes in SUM190, SUM149 and MDA-MB-231 cells after exposure to the supernatants (SNs) for 4 days. Data represent means±S.D., *n*=3; *P<0.05 compared with vehicle-supernatants group. See also Supplementary Figure 1

To determine whether autocrine mechanisms are implicated in the tumor microenvironment after intermittent exposure to chemotherapeutic drugs and enhance breast CSC phenotypes, we treated breast cancer cells with vehicle or chemotherapeutic drugs (paclitaxel, 5-fluorouracil or doxorubicin) for 4 days, followed by washes, medium change and continual culture in fresh medium in the absence of any drug for an additional 4 days. At day 8, the drug-free and cell-free supernatants were collected and used for culture of the same line of breast cancer cells. The supernatants derived from cells receiving control vehicle-treatment were denoted as vehicle-derived supernatants and from cells receiving paclitaxel treatment as paclitaxel-derived supernatants (Figure 1a).

After exposure of untreated cells to paclitaxel-derived supernatants for 4 days, both CD44^high^/CD24^-/low^ and ALDH-positive subpopulations in SUM149 and MDA-MB-231 cells (triple negative breast cancer – TNBC), and SUM190 (inflammatory breast cancer) cells increased significantly by 2-3 fold (Figure 1b; Supplementary Figure 1, flow cytometry). A marked upregulation of ALDH1, CD44 and OCT4 proteins were also observed (Figure 1c, western blot). As CD44^high^/CD24^-/low^ and/or ALDH1 have been commonly used in the characterization of breast CSCs,^16, 19, 20, 21^ these results suggest that some factors produced by breast cancer cells themselves (i.e., autocrine factors) after drug withdrawal facilitate CSC enrichment.

As a functional measure of CSC properties, we performed mammosphere assays based on the ability of breast CSCs to generate multicellular spheroids in suspension culture. SUM149, MDA-MB-231and SUM190 cells were treated for 4 days with vehicle- or paclitaxel-derived supernatants, and reseeded in an ultra-low 6-well plate (2000 cells/well) for an additional 8 days. Cells pretreated with paclitaxel-derived supernatants formed ~2–3 folds more mammospheres and also generated larger spheroids than cells cultured in vehicle-derived supernatants (Figure 1d). The increased mammosphere forming efficiency suggests that the autocrine factors in paclitaxel-derived supernatants enhance the self-renewal capacity of CSCs.

To further characterize CSC phenotype, we assessed gene expression profiles. The qPCR results showed that treatment with paclitaxel-derived supernatants resulted in a marked upregulation of stem cells-associated genes *ALDH1*, *SOX2*, *c-MYC*, *OCT4* and *NANOG* (Figure 1e). In addition, epithelial–mesenchymal transition (EMT)-related genes *SLUG*, *MMP9*, *ZEB1* and *SNAIL* were also elevated, while E-cadherin, an epithelial cell marker, were reciprocally decreased in all three breast cancer cell lines examined (Figure 1e). As expected, after exposure to different proportions of paclitaxel-derived supernatants for 4 days, breast cancer cells became less susceptible to subsequent paclitaxel killing in a dose-dependent manner (Supplementary Figure 2A). Moreover, pre-exposure to paclitaxel-derived supernatants also led to reduced apoptosis in bulk and CSC populations while increased CSCs (both CD44^high^/CD24^-/low^ and ALDH^+^ subpopulations) in response to paclitaxel treatment (Supplementary Figures 2B–D and 3, flow cytometry). Collectively, these data indicate that autocrine factors produced by breast cancer cells themselves after chemotherapy withdrawal lead to the induction of CSC properties and chemoresistance.

### Chemotherapeutic drug treatment stimulates breast cancer cells to secret inflammatory cytokines that activate inflammatory-related pathways

**Figure 2:**
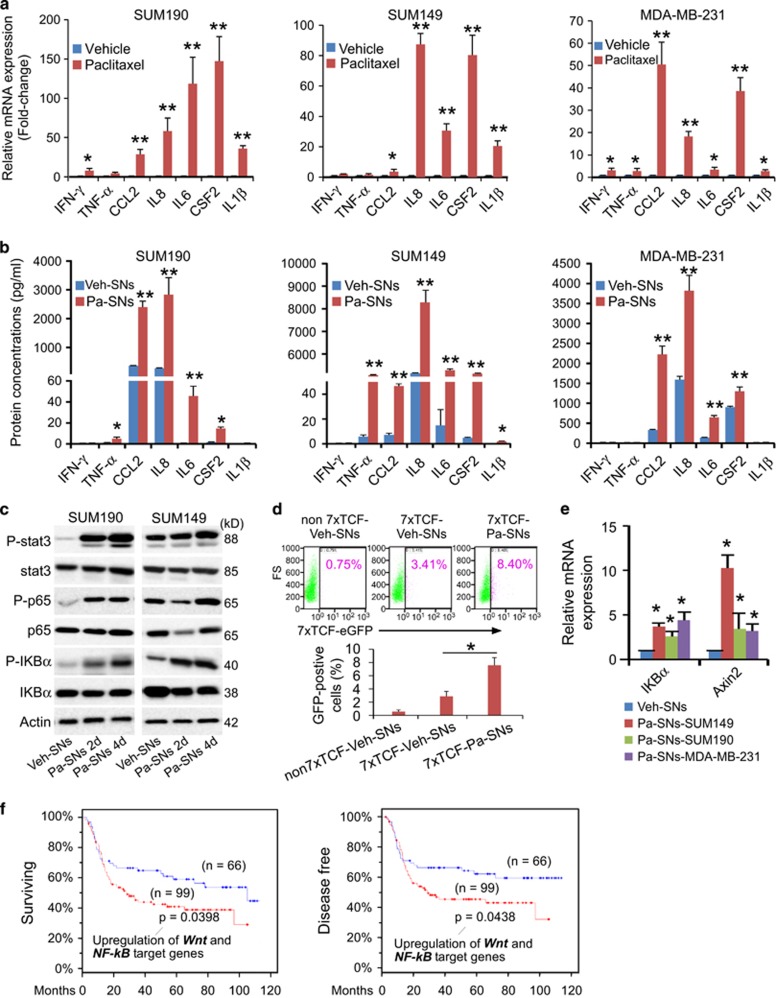
Chemotherapeutic drug stimulates breast cancer cells to secret inflammatory cytokines to activate inflammatory-related pathways. (**a**) qPCR analysis of the gene expression of various cytokines and chemokines in the cells after treatment for 4 days with vehicle or paclitaxel (15 nM) followed by culture in fresh medium for additional 4 days. At day 8, after 4-day paclitaxel withdrawal, cells were harvested for qPCR analysis (the culture media were collected as supernatants analyzed by human cytokine arrays in B or applied to fresh cells in consequent experiments). Data from vehicle-treated cells were set as 1 and *GAPDH* mRNA was used to normalize variability in template loading. Data represent the means±S.D., *n*=4; *P<0.05, **P<0.001. (**b**) Human cytokine arrays for protein concentrations of IFN-*γ*, TNF-*α*, CCL2, IL6, IL8 and IL1*β* in the supernatants as described in **a**. Analyses were performed blindly by Eve Technologies using human cytokine multiplex assays. Data represent means±S.D., *n*=3; *P<0.05, **P<0.001. Veh-SNs: supernatants (SNs) derived from vehicle (Veh)- pretreated cells; Pa-SNs: derived from paclitaxel (Pa, 15 nM)-pretreated cells. (**c**) Western blotting analysis of the phosphorylation of NF-*κ*B, I*κ*B*α* and Stat3 in SUM190 and SUM149 cells at 2 and 4 days after exposure to the vehicle (Veh)-derived or paclitaxel (Pa, 15 nM)-derived supernatants (SNs). *β*-actin as an internal loading control. (**d**) Flow cytometric analysis of the 7xTCF-eGFP reporter activity in Wnt reporter subline 7xTCF-SUM190 in the presence of vehicle (Veh)-derived or paclitaxel (Pa)-derived supernatants (SNs) for 4 days. Mock transduced SUM190 cells (non7xTCF) and 7xTCF-SUM190 subline generated by lentiviral transduction, and denoted as non7xTCF-Veh-SNs, 7xTCF-Veh-SNs and 7xTCF-Pa-SNs. Live cells were pre-gated based on 7-AAD-negative. The TCF-eGFP reporter activity was measured by the percentage of GFP-positive cells after different treatments. Data represent the means±S.D., *n*=3, *P<0.05. (**e**) qPCR analysis of the expression of NF-*κ*B target gene *IKBA* and Wnt target gene *AXIN2* in SUM190, SUM149 and MDA-MB-231 cells after exposure to vehicle (Veh)-derived or paclitaxel (Pa, 15 nM)-derived supernatants (SNs) for 4 days. Data represent means±S.D., *n*=3; *P<0.05. (**f**) Kaplan–Meier survival analysis of overall survival and disease-free survival time in TNBC patients treated with chemotherapeutic drugs. Poor survival rate and shorter disease-free survival time were observed if patients’ tumor samples expressed high levels of Wnt and NF-*k*B target genes (cBioPortal, TCGA, Nature Communications 2016, mRNA microarray, z-score±2.00, patient set: 165 TNBC patients treated with chemotherapy)

Since inflammatory cytokines have been closely associated with cancer progression and CSC development,^22^ we asked whether paclitaxel-derived supernatants possess high levels of inflammatory cytokines that led to CSC enrichment. We found that, after 4-day paclitaxel withdrawal, the gene expression levels of cytokine/chemokine in SUM190, SUM149 and MDA-MB-231 cells remained extremely high (Figure 2a). In particular, the gene expression levels of *IL6*, *IL8*, *CSF2* and *CCL2* were significantly elevated. Consistently, the protein levels of these cytokines/chemokines in supernatants were also markedly increased (e.g., up to 80-fold greater for IL8) as measured by multiple human cytokines assays (Figure 2b), indicating robust production of cytokine proteins by breast cancer cells themselves after exposure to chemotherapeutics followed by drug withdrawal.

The high levels of cytokine proteins in supernatants produced by breast cancer cells after paclitaxel withdrawal prompted us to examine the status of several inflammation-associated pathways, including signal transducer and activator of transcription 3 (STAT3), nuclear factor-kappa-B (NF-*κ*B) and Wnt/*β*-catenin signaling pathways. As shown in Figure 2c, exposure to paclitaxel-derived supernatants (autocrine factors) for 4 days resulted in a significant increase in the expression of phosphorylated NF-*κ*B, I*κ*B*α* and STAT3 proteins in the same line of breast cancer cells. In addition, a significant increase in Wnt reporter activity was observed in 7xTCF-transduced cells after culture with paclitaxel-derived supernatants for 4 days (Figure 2d). The 7xTCF is a real-time fluorescent Wnt reporter construct containing 7 tandem Tcf/Lef consensus binding sites upstream of a minimal promoter driving expression of GFP and an SV40-puromycin selection cassette.^23^ Consistently, both NF-*κ*B and Wnt/*β*-catenin target genes were significantly upregulated (Figure 2e). These results suggest that breast cancer cell-produced supernatant-factors (autocrine factors) can activate both NF-*κ*B and Wnt/*β*-catenin pathways of the same line of breast cancer cells.

After analysis of the METABRIC data set,^24^ we found that among TNBC patients treated with chemotherapeutic drugs, samples with increased expression of Wnt/*β*-catenin target genes and NF-kB target genes exhibited poor survival rate and shorter disease-free time as compared with samples that expressed lower levels of these genes (Figure 2f). Together, our *in vitro* results and the clinical data analysis suggest that chemotherapeutic treatment may lead to enhanced secretion of inflammatory cytokines that can activate inflammatory pathways in the same line of breast cancer cells, which correlates positively with enhanced CSC phenotypes and poor clinical outcomes.

### Activation of *β*-catenin and NF-*κ*B pathways by autocrine factors enhances production of inflammatory cytokines, forming a forward-feedback loop to promote further enrichment of CSCs

The NF-*κ*B signaling pathway is known to play a critical role in the induction and maintenance of inflammatory cytokines.^6, 7, 25, 26^ Thus, consideration was given to the possibility that activation of NF-*κ*B initiated by paclitaxel-induced supernatants enabled breast cancer cells to continually produce inflammatory cytokines, thereby forming an autocrine inflammatory forward-feedback loop that further promotes CSC enrichment. In addition, whether or not co-activation of Wnt/*β*-catenin and NF-*κ*B pathways is necessary in the above process was determined by using a loss-of-function approach.

**Figure 3:**
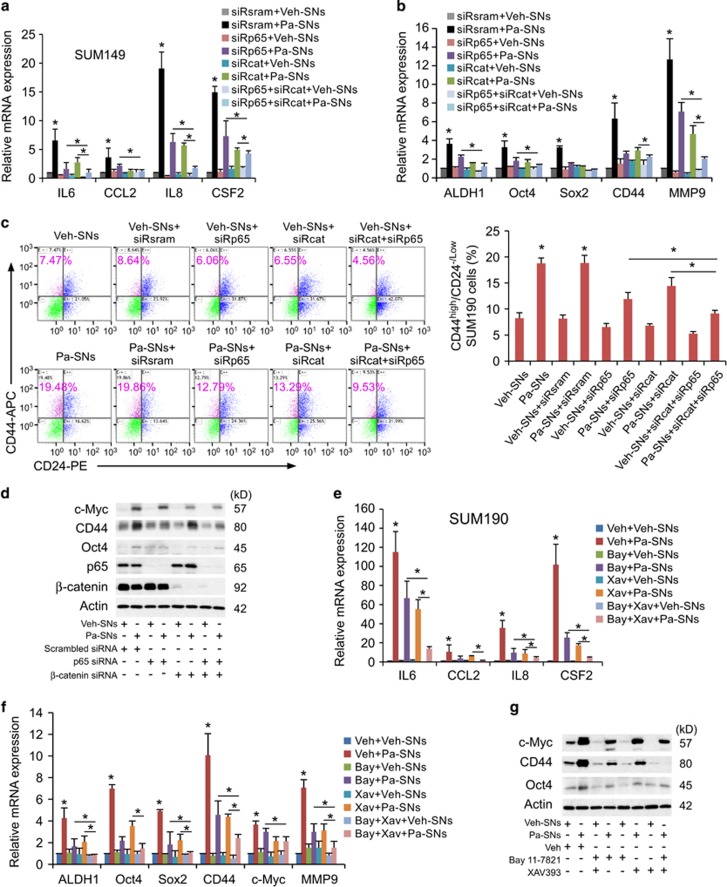
Activation of Wnt/*β* -catenin and NF-*κ*B pathways by the factors produced by breast cancer cells enhances the production of inflammatory cytokines from the same cell line, forming a forward-feedback loop to further promote CSC enrichment. (**a-d**). Knockdown of NF-*κ*B (p65) and/or *β*-catenin markedly abolishes the upregulated cytokine genes, stem cell-associated genes (**a** and **b**, qPCR), CD4^4high^/CD2^4-/low^ subpopulation (**c**, flow cytometry) and stem cell-associated proteins (**d**, western blot) after exposure to the paclitaxel-derived supernatants generated by the same cell line. Western blot also showed an almost complete knockdown of NF-*κ*B p65 and *β*-catenin expression after transfection of NF-*κ*B p65 and *β*-catenin siRNA with *β*-actin as an internal loading control. SUM149 cells were transfected with siRNA oligos against NF-*κ*B p65 (siRp65), *β*-catenin (siRcat) or non-targeting oligos (siRsram) for 24 h, and then treated with vehicle (Veh)- or paclitaxel (Pa)-derived supernatants (SNs) for 4 days, followed by qPCR, flow cytometric and western blot analyses. Data represent means±S.D., *n*=3; *P<0.05. (**e-g**) Similar results are obtained by using small molecule inhibitors. Inhibition of NF-*κ*B and *β*-catenin pathways effectively diminishes the upregulated expression of cytokine genes and stem cell-related genes (**e** and **f**, qPCR), and stem cell-related proteins (**g**, western blot with *β*-actin as an internal loading control) induced by paclitaxel-derived supernatants (Pa-SNs). SUM190 cells were pretreated for 2 h alone or in combination with vehicle (Veh), Bay 11-7821 (Bay, 5 *μ*M, a NF-*κ*B inhibitor) and/or XAV939 (Xav, 10 *μ*M, a Wnt/*β*-catenin inhibitor), followed by exposure to vehicle (Veh)- or paclitaxel (Pa)-derived supernatants (SNs) for 4 days in the presence or absence of the same vehicle or inhibitors. Data represent averages±S.D., *n*=3; *P<0.05. See also Supplementary Figures 4 and 5

Significantly, siRNA knockdown of *β*-catenin and/or NF-*κ*B p65 markedly diminished the upregulation of cytokine genes initiated by the exposure to paclitaxel-derived supernatants produced by the same line of breast cancer cells (Figure 3a). In addition, knockdown of NF-*κ*B and/or Wnt/*β*-catenin significantly suppressed stemness genes and abolished CD44^high^/CD24^−/low^ enrichment induced by paclitaxel-derived supernatants (Figures 3b and c). Consistently, the expression levels of CSC marker proteins CD44, OCT4 and c-MYC were also significantly suppressed after siRNA knockdown (Figure 3d, western blot), suggesting that both Wnt/*β*-catenin and NF-*κ*B pathways are required. The knockdown efficiency had been confirmed by the significant reduction of NF-*κ*B and Wnt target genes (Supplementary Figures 4E and F) and p65 and *β*-catenin proteins (Figure 3d).

Similar results were obtained by using small molecule inhibitors. Blockade of Wnt/*β*-catenin with XAV939 and NF-*κ*B with Bay-11-7821 robustly suppressed the expression of inflammatory cytokine genes, CSC-associated genes and proteins (Figures 3e–g), CD44^high^/CD24^−/low^ CSC subpopulation and cell migration (Supplementary Figure 5). To a certain degree, these data are consistent with those shown in Figures 1 and 2 where similar results were obtained from three different breast cancer cell lines. As suppression of STAT3 signaling pathways with a special small molecule inhibitor did not show significant effects (data not shown), it suggests that co-activation Wnt/*β*-catenin and NF-*κ*B has an important role in the aforementioned biological processes.

Collectively, these data suggest that tumor cell-produced inflammatory cytokines activate both Wnt/*β*-catenin and NF-*κ*B pathways of the same line of tumor cells, which in turn enhance inflammatory cytokine production, forming an autocrine forward-feedback loop to promote CSC enrichment after chemotherapy withdrawal. It seems that formation of autocrine inflammatory forward-feedback loop is a conserved response to chemotherapeutic drugs. Similar to paclitaxel-derived supernatants, doxorubicin-derived supernatants or 5-fluorouracil-derived supernatants also increased gene expression of *IL8* and *IL6*, enhanced I*κ*B*α* phosphorylation and 7xTCF-eGFP reporter activity (indicating activation of NF-kB and Wnt pathways), upregulated the expression of CSC-associated c-MYC protein, and augmented expression of CSC-associated genes *ALDH1*, *OCT4*, *SOX2* and *c-MYC* (Supplementary Figures 4A–D).

### Inhibition of IL8 or its receptors diminishes CSC properties induced by autocrine inflammatory forward-feedback loop

**Figure 4:**
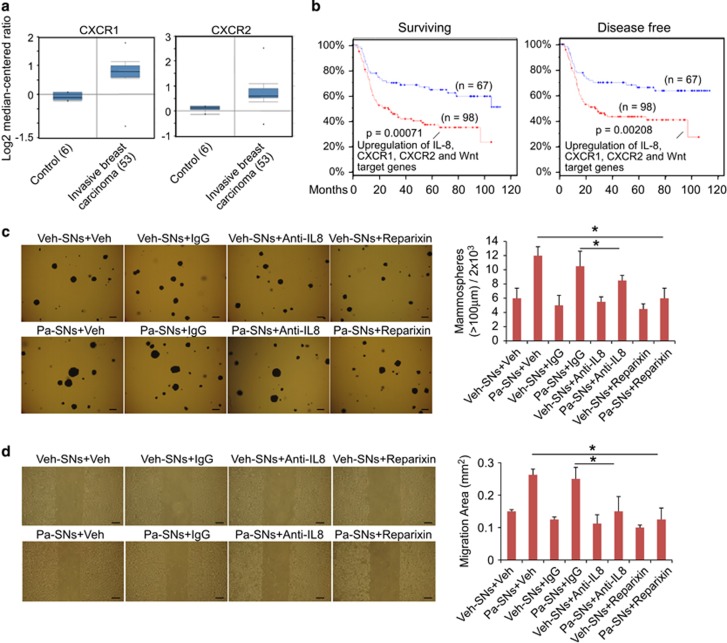
Inhibition of IL8 or its receptors diminishes CSC properties induced by autocrine inflammatory forward-feedback loop. (**a**) A significant increase in the expression of IL8 receptors CXCR1 and CXCR2 in the invasive breast carcinoma samples in comparison to normal control (Oncomine data set repository, human breast cancer data sets, www.oncomine.org). (**b**) Kaplan–Meier analysis of overall survival and disease-free survival time in TNBC patients treated with chemotherapeutic drugs. TNBC patients treated with chemotherapeutic drugs exhibit poor survival rate and shorter disease-free survival time if their tumor samples expressed high levels of *IL8*, *CXCR1*, *CXCR2* genes and Wnt target genes (*CD44*, c-*MYC*, *SOX2*, *HNF1A*, and *PPARD*, cBioPortal, TCGA, Nature Communications 2016, mRNA microarray, z-score±2.00). (**c**) Soft-agar colony formation assay to evaluate tumorigenic potential. SUM149 (5 × 10^3^ cells/each well, 12-well plate) were seeded in soft agar for 17 days and cultured in vehicle (Veh)-derived or paclitaxel (Pa)-derived supernatants (SNs) in the presence of vehicle, control antibody (IgG), anti-IL8 antibody (Anti-IL8) or reparixin (a CXCR1/2 inhibitor, 10 *μ*M). Cellular aggregates with diameter of greater than 100 *μ*m were counted as colonies after staining with MTT for live cells. Similar results were obtained from SUM190 and MDA-MB-231 breast cancer cells. Scale bar, 100 *μ*m. Data represent means±S.D., *n*=4; *P<0.05. (**d**) Anti-IL8 antibody or CXCR1/2 inhibitor reparixin suppresses migration of SUM149 cells induced by paclitaxel-derived supernatants in wound-healing assays. Cells were grown to confluence, scratched, and then observed and quantified for wound-healing migration after exposure to vehicle (Veh)- or paclitaxel (Pa)-derived supernatants (SNs) in presence of vehicle (Veh), control antibody IgG, anti-IL8 or Reparixin. Representative scratch healing images are shown. Histogram represents migration (in mm^2^) over 24 h after scratch. Scale bar, 100 *μ*m. Data represent means±S.D., *n*=4; *P<0.05

Given that inhibition of NF-*κ*B and Wnt/*β*-catenin pathways effectively blocked inflammatory autocrine forward-feedback loop, we next asked whether this forward-feedback loop could also be impeded by blocking key cytokines and/or their receptors (part of the upstream loop). As increases in IL8 protein levels after treatment with paclitaxel-derived supernatants were greatest in all three breast cancer cell lines (Figure 2b), we postulated that blockade of IL8 or its receptor would diminish the autocrine forward-feedback loop and CSC development. This possibility was supported by significant upregulation of IL8 receptors (CXCL1 and CXCL2) in clinical invasive breast cancer samples but not in normal controls (Figure 4a). After further analyzing the recently available clinical data set containing chemotherapeutic information (breast cancer TCGA, cBioPortal, Nature Communications 2016), we found that TNBC patients treated with chemotherapeutic drugs exhibited poor survival rate (66.7 *vs* 282.8 months, *P*=0.00071) and shorter disease-free survival time (*P*=0.00208) if tumor samples expressed higher levels of *IL8*, *CXCR1*, *CXCR2* and the Wnt target genes than those expressed lower levels (Figure 4b). These clinical data suggest a tangible correlation between IL8/IL8 receptors, Wnt target genes and breast cancer progression after chemotherapy.

To determine whether IL8 has a causative role in the formation of inflammatory autocrine forward-feedback loop, we performed tumorsphere formation and wound scratch assays, two functional assessments commonly used in the study of CSC self-renewal and cancer cell migration/invasion.^25, 26, 27^ Breast cancer cells were cultured in vehicle- or paclitaxel-derived supernatants in the presence of anti-IgG control antibody, anti-IL8 antibody, vehicle or CXCR1/2 inhibitor for 4 days, followed by assessment of tumorsphere-forming or scratch-migration capacity. As anticipated, blockade of IL8 using a neutralizing antibody significantly decreased tumorsphere formation induced by paclitaxel-derived supernatants (Figure 4c). Pharmacological inhibitor reparixin capable of suppressing IL8 receptor CXCR1 and CXCR2 also markedly reduced tumorsphere formation (Figure 4c). In addition, quantification of wound width at 24-hour post injury revealed that anti-IL8 antibody or reparixin significantly inhibited tumor cell migration induced by paclitaxel-derived supernatants (Figure 4d). These results suggest that, in addition to NF-*κ*B and Wnt/*β*-catenin pathways, IL8 and IL8 receptors are important components in the formation of autocrine inflammatory forward-feedback loop to promote CSC properties after chemotherapy withdrawal.

### Paclitaxel withdrawal followed by blockade of autocrine inflammatory forward-feedback loop prevent CSC enrichment *in vitro*

**Figure 5:**
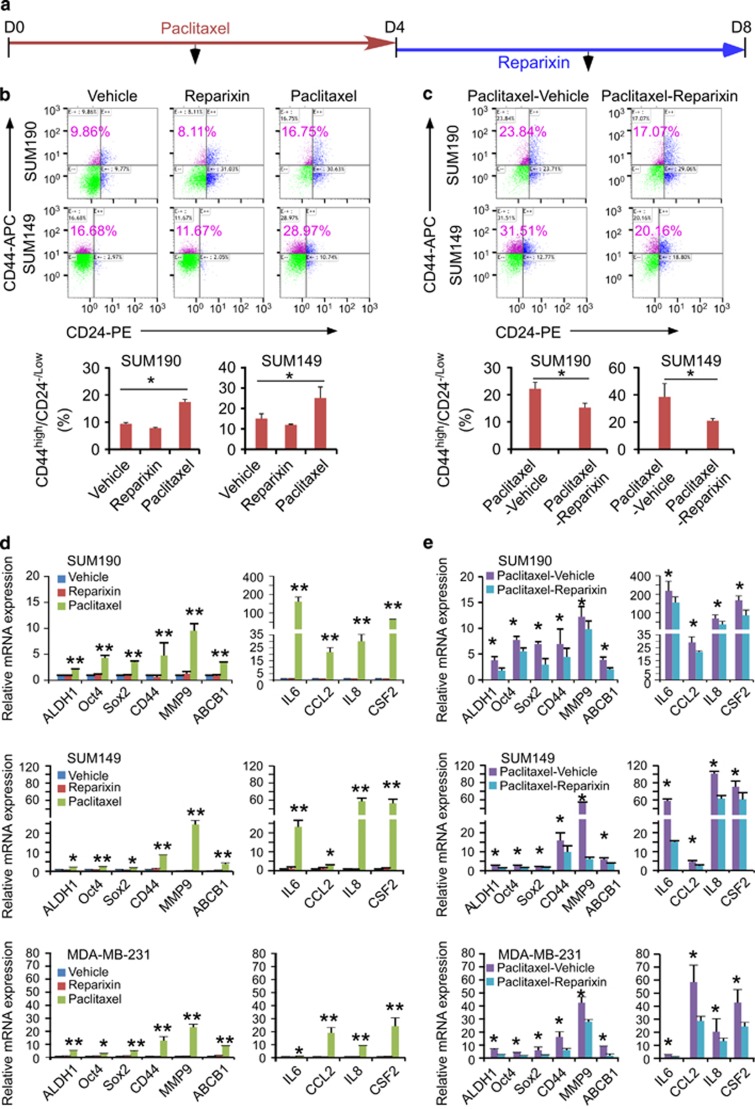
Paclitaxel withdrawal followed by CXCR1/2 inhibition prevents CSC enrichment *in vitro.* (**a**) A scheme of culture protocol used to study the effects of paclitaxel treatment followed by reparixin inhibition on breast cancer cells. SUM190, SUM149 and MDA-MB-231 cells were treated with paclitaxel (15 nM) for 4 days, washed with sterilized PBS, changed to fresh medium in the presence or absence of reparixin for 4 days (days 5-8). (**b** and **c**) Percentage of CD44^high^/CD24^-/low^ cells in SUM190 and SUM149 was assessed by flow cytometry at day 4 after paclitaxel treatment as described in **a** (**b**) and at day 8 after reparixin treatment (**c**). Data represent means±S.D., *n*=3; *P<0.05. (**d** and **e**) qPCR analysis of the indicated stemness- and cytokine-related genes in SUM190, SUM149 and MDA-MB-231 cells after treatment with paclitaxel at day 4 (**d**), followed by reparixin treatment at day 8 (**e**). Data represent means±S.D., *n*=3; *P<0.05

As inhibition of IL8 receptor CXCR1/2 with reparixin diminished CSC tumorsphere forming and cancer cell migration induced by autocrine inflammatory forward-feedback loop (Figures 4c and d), we then asked whether reparixin would inhibit CSC enrichment after paclitaxel withdrawal *in vitro*. We treated breast cancer cells with paclitaxel for 4-day followed by reparixin for an additional 4-day in the absence of paclitaxel (Figure 5a). The CD44^high^/CD24^-/low^ subpopulation was analyzed by flow cytometry at day 4 immediately after paclitaxel withdrawal (Figure 5b) and at day 8 after treatment with vehicle or reparixin (Figure 5c). We found that CD44^high^/CD24^-/low^ subpopulation treated with paclitaxel followed by vehicle was increased ~2.5-fold at day 8 (Figure 5c) as compared with that treated with vehicle or reparixin alone, and 1.5-fold as compared with that treated with paclitaxel alone at day 4 (Figure 5b), suggesting continual enrichment of CSC subpopulation after paclitaxel removal. In sharp contrast, breast cancer cells treated with reparixin after paclitaxel withdrawal markedly reduced CSCs subpopulation in comparison to vehicle-treated group at day 8 or paclitaxel alone treated group at day 4 (Figures 5b and c, flow cytometry).

Analysis of gene expression profiling showed that expression of cytokines- and EMT-related genes *IL6*, *IL8*, *CCL2*, *CSF2, MMP9* and *ABCB1* as well as stem cell-associated genes *ALDH1*, *SOX2*, *OCT4* and *CD44* was continuously increased at day 8 after paclitaxel withdrawal for 4 days, and inhibited by reparixin after removal of paclitaxel (Figures 5d and e, qPCR). Thus, reparixin treatment can hamper autocrine inflammatory forward-feedback loop and CSC enrichment *in vitro*.

### Paclitaxel withdrawal followed by blockade of autocrine inflammatory forward-feedback loop diminishes tumor mass and repopulation of drug-resistant breast cancer cells/CSCs *in vivo*

**Figure 6:**
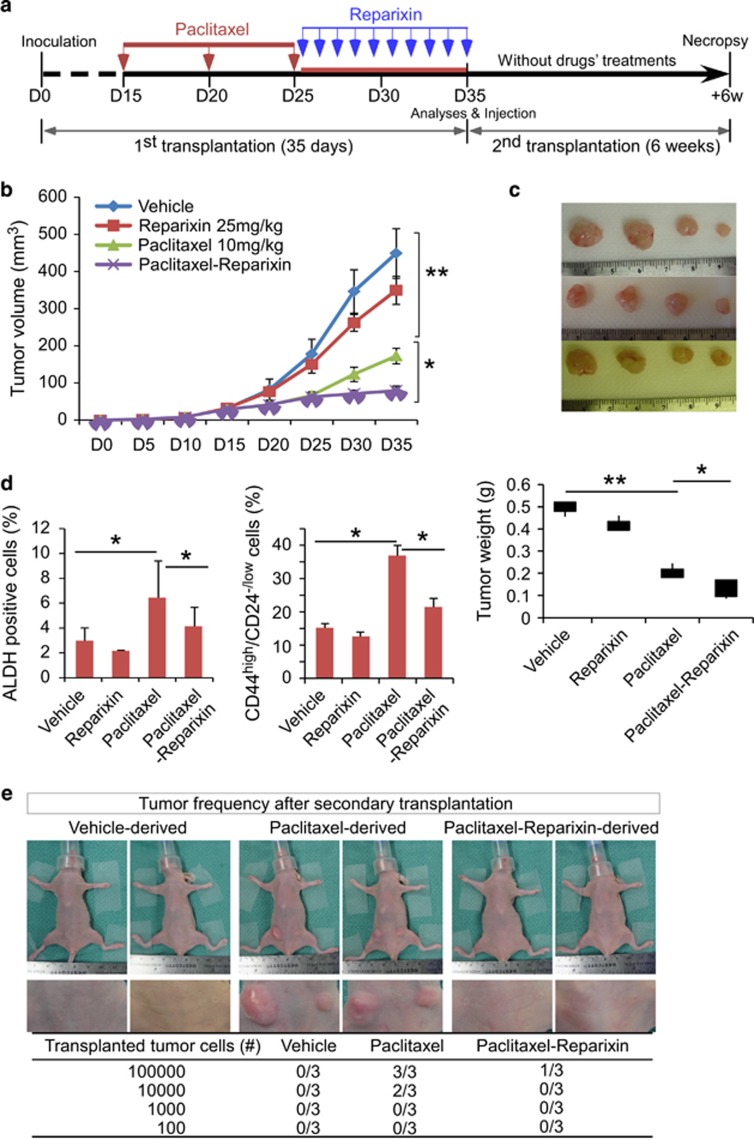
Paclitaxel withdrawal followed by reparixin administration diminishes tumor mass and repopulation of drug-resistant breast cancer cells/CSCs *in vivo.* (**a**) A schematic representation of the experimental protocol as described in Materials and Methods. (**b**) SUM149 breast cancer cells were implanted into the mammary fat pads of athymic nude mice. When the tumors reached a mean diameter of 4 mm (day 15), mice were randomized into 4 groups and intraperitoneally injected with vehicle, paclitaxel alone (10 mg/kg at days 15, 20 and 25), reparixin alone (25 mg/kg every day starting from day 26 for 10 days), or paclitaxel followed by reparixin. Tumor volumes were measured as described in Materials and Methods. Data represent means±S.D.; *n*=4–8 mice for each group; *P<0.05, **P<0.001. (**c**) Smaller tumor sizes were observed in mice treated with paclitaxel followed by reparixin compared with paclitaxel and reparixin alone. Data represent means±S.D., *n*=4–8 mice; *P<0.05. (**d**) The percentage of CD44^high^/CD24^-/low^ cells and ALDH-positive cells in tumors was determined by flow cytometry. Paclitaxel injection retards tumor growth but also significantly increases CSC pool. In contrast, paclitaxel treatment followed by reparixin injection abrogates paclitaxel-enriched CSCs. Data represent means±S.D., *n*=3; *P<0.05. (**e**) Photos showing mice and tumors and table showing the tumor frequency after secondary transplantation. SUM149 xenografts harvested from the mice that had been injected with vehicle, paclitaxel or paclitaxel followed by reparixin after the first tumor cell transplantation were dissociated into single-cell suspensions and re-transplanted into the mammary fat pad of new nude mice in serial limiting dilutions (10^5^, 10^4^, 10^3^ or 10^2^ cells per injection). Tumor formation was observed for 6 weeks following inoculation and tumor frequency was significantly reduced after paclitaxel withdrawal followed by reparixin injection

To determine whether blockade of autocrine inflammatory forward-feedback loop after paclitaxel withdrawal exhibits similar effects *in vivo*, we transplanted SUM149 cells into the mammary fat pads of athymic nude mice. When the tumor reached a mean diameter of 4 mm after 15 days of implantation, mice were randomized into 4 groups and injected intraperitoneally with vehicle, paclitaxel (10 mg/kg on days 15, 20 and 25), IL8 receptor CXCR1/2 inhibitor reparixin (25 mg/kg once every other day starting from days 26 for 10 days), or paclitaxel following by reparixin (Figure 6a). To determine CSC pool *in vivo*, we harvested tumors at the end of the treatment (at day 35) and assessed CD44^high^/CD24^-/low^ subpopulation and ALDH-positive population using flow cytometry. As shown in Figures 6b–d, in comparison to vehicle control, paclitaxel treatment alone increased CSC subpopulation over 2-fold after paclitaxel withdrawal for 10 days (*P*<0.05), although it impeded tumor growth (*P*<0.05), consistent with *in vitro* findings that chemotherapy withdrawal enriched CSCs. While reparixin treatment alone only showed a moderate effect on tumor growth, it slightly suppressed CSC enrichment. Notably, reparixin administration after paclitaxel withdrawal markedly reduced tumor burden compared with all other groups, and also inhibited paclitaxel treatment-induced CSC enrichment. Reparixin administration following conventional chemotherapy, therefore, effectively inhibits both bulk tumor and tumorigenic drug-resistant cancer cells and CSCs.

To determine whether tumors containing drug-resistant breast cancer cells and/or increased CD44^high^/CD24^-/low^ fractions possess greater tumor-initiating potential, we performed secondary transplantation. We serially diluted tumor cells containing various percentage of CD44^high^/CD24^-/low^ populations isolated from primary tumors, and transplanted them into athymic nude mice without further treatment. Significantly, tumor cells isolated from paclitaxel-treated mice exhibited a greater tumor-initiating capacity. On the contrary, tumor cells isolated from reparixin administration after paclitaxel withdrawal had markedly reduced tumor-initiating capacity (Figure 6e). Based *in vitro* and *in vivo* results, our data indicate that inhibition of autocrine inflammatory forward-feedback loop after chemotherapy withdrawal enhances drug efficacy by reducing tumor burden and enrichment of drug-resistant breast cancer cells and CSCs.

## Discussion

Chemotherapy is a standard of care in clinical oncology today due to its effectiveness in reducing tumor burden and improving overall survival. Most chemotherapeutic drugs are administered at intervals to reduce severe side effects.^3, 4, 5^ A critical question that remains unanswered is whether drug resistance is associated with intermittent drug administration. If so, what are the underlying mechanisms and effective approaches to prevent it? Our results indicate that after paclitaxel withdrawal, breast cancer cells produce inflammatory cytokines to activate both Wnt/*β*-catenin and NF-*κ*B signaling pathways which in turn further promote cytokine production from breast cancer cells (Figures 1, 2 and 3). Formation of an autocrine inflammatory forward-feedback loop after termination of chemotherapy leads to repopulation of drug-resistant breast cancer cells/CSCs (Figure 6).

It has been suggested that breast cancer cells produce cytokines and/or growth factors to facilitate their survival and expansion. In the present studies, we demonstrate that secretion of CCL2, IL8, IL6 and CSF2 are markedly increased after paclitaxel withdrawal (Figure 2). After termination of paclitaxel treatment for several days, breast cancer cells continually produce higher levels of cytokines to promote CSC enrichment (Figures 2,3,4 and 5). An autocrine forward-feedback loop involving the released inflammatory cytokines and intracellular signaling pathways may take place during intermittent administration of chemotherapeutic drugs in typical clinical settings.

Indeed, after removal of paclitaxel, culture supernatants enriched with inflammatory cytokines produced by breast cancer cells markedly enhance phosphorylation of NF-*κ*B (Figure 2). Cytokine signaling is known to activate NF-*κ*B, and NF-*κ*B signaling is known to promote cytokine production.^28, 29^ However, inhibition of NF-*κ*B signaling alone is insufficient to increase sensitivity to cytotoxic drugs^30^ and was ineffective at diminishing cytokine production from breast cancer cells (Figure 3). We revealed that activation of Wnt/*β*-catenin and NF-*κ*B is important for the formation of such an autocrine inflammatory forward-feedback loop. As a result, blockade of Wnt/*β*-catenin and NF-*κ*B effectively abrogates the cytokine feedback loop, diminishes the expression of stemness genes and the enrichment of CSC subpopulation (Figure 3). Co-activation of NF-*κ*B and Wnt/*β*-catenin pathways has recently been shown to be essential for the development of breast CSCs in response to toll-like receptor 3 stimulation,^25^ and also have a cooperative role in conferring CSC properties in an intestinal cancer model.^31^ In addition, phosphorylated NF-*κ*B has been shown to bind to *β*-catenin via CREB-binding protein to strengthen *β*-catenin transcriptional activities, resulting in generation of strong self-renewal signaling in myeloid leukemia stem cells.^32^ This suggests that inhibition of Wnt/*β*-catenin and NF-*κ*B pathways after chemotherapy withdrawal will maximize clinical efficacy.

We also identify IL8 as an alternative target for blockade of autocrine inflammatory forward-feedback loop after chemotherapy withdrawal (Figures 2 and 4). IL8 signal, via two cell surface G-protein–coupled receptors CXCR1 and CXCR2, has been shown to be upregulated in several types of cancer including breast cancer and associated with increased CSC pool *in vitro* and poor prognosis in patients.^33, 34, 35^ Our *in vitro* data demonstrated that blockade of IL8 with a neutralizing antibody or blockade of IL8 receptors CXCR1 and CXCR2 with reparixin inhibits autocrine inflammatory forward-feedback loop triggered by paclitaxel treatment (Figures 4 and 5). Our *in vivo* data also show that administration of reparixin after paclitaxel withdrawal markedly reduced tumor mass and diminished drug-resistant breast cancer cells/CSC subpopulation, subsequently diminishing tumor-initiating capacity (Figure 6). Consistently, patients with TNBC treated with chemotherapeutic drugs exhibited very poor survival rate and shorter disease-free survival time if their tumor samples expressed high levels of IL8, CXCR1/2 and Wnt signals (Figure 4b).

In our *in vitro* experiments, inhibition of NF-*κ*B and/or Wnt signaling pathways (with siRNA or small molecules) exhibits better potency than neutralization of IL8 with antibody or inhibition of CXCR1/2 with reparixin in the blockade of stem cell- and EMT-associated gene expression. Further optimization of the inhibitory dosage of IL8 antibody and reparixin seems to be required. It is also possible that other undefined factors may have cooperative roles, warranting further studies.

In conclusion, our studies identify an autocrine inflammatory forward-feedback loop after withdrawal of chemotherapeutic drugs, which leads to the repopulation of drug-resistant breast cancer cells/CSCs and facilitate tumor progression. Our results suggest that inhibition of NF-*κ*B and Wnt/*β*-catenin pathways or key cytokine signals along autocrine inflammatory forward-feedback loop after chemotherapy withdrawal will reduce tumor mass and secondary tumor initiation. Given that NF-*κ*B and Wnt/*β*-catenin inhibitors and reparixin have been approved by FDA for clinical trials, administration of these reagents between intermittent cycles of chemotherapy may reduce toxicity, and are worth studying in the context of future clinical trials to reduce disease relapse and improve patient outcomes.

## Materials and Methods

### Cell culture and reagents

SUM149 and SUM190 breast cancer cells were obtained from Asterand (Detroit, MI, USA) and cultured in Hams F12 media (Mediatech, Manassas, VA, USA) containing 5 *μ*g/ml insulin, 1 *μ*g/ml hydrocortisone, 10 mM HEPES and antibiotics (penicillin/streptomycin). Medium for SUM149 cells was further supplemented with 5% fetal bovine serum (HyClone, Logan, UT, USA), and for SUM190 cells was further supplemented with 5 mM ethanolamine, 5 *μ*g/ml transferrin, 6.6 ng/ml 3,3',5-triiodo-l-thyronine sodium salt, 8.7 ng/ml sodium selenite, and 1 mg/ml bovine serum albumin. Cells were cultured at 37 °C in a 5% CO_2_ incubator. Breast cancer cell line MDA-MB-231 was purchased from the American Type Culture Collection (Manassas, VA, USA) and maintained in DMEM-F12 (1:1) supplemented with 10% Fetal Bovine Serum. Paclitaxel, insulin, hydrocortisone, HEPES, and bovine serum albumin were purchased from Sigma-Aldrich (St. Louis, MO, USA). Reparixin was purchased from MedChemExpress (MedChemExpress Biotechnology, Monmouth, NJ, USA).

### Flow cytometry analysis

Cancer cells dissociated from the transplanted tumor tissues or from culture plates were counted and re-suspended in 100 *μ*l of HBSS (Gibco, Langley, OK, USA) containing 2% heat-inactivated fetal bovine serum (FACS buffer) and 10^5^ cells. Five microliters of mouse IgG solution (1 mg/ml) was added and incubated on ice for 10 min. According to the manufacturer’s recommendation, appropriate antibodies were added and incubated for 30 min on ice. The cells were then washed twice with FACS buffer and re-suspended in 0.2 ml of FACS buffer that contained 7-aminoactinomycin D (7-AAD, eBioscience, San Diego, CA, 1 *μ*g/ml, final concentration) to exclude dead cells. Antibodies used were anti-CD44 (APC) and anti-CD24 (PE), which were purchased from BD Pharmingen. ALDH activity was examined with the ALDEFLUOR kit (Stem cell Technologies, Vancouver, Canada). Cell apoptosis was assessed using an Annexin V-PE-Cy7 Apoptosis Detection Kit (eBiosciences, Burlington, ON, Canada) for Beckman Cyan-ADP 9 flow cytometer or V450 Annexin V (BD) for BD LSR Fortessa. Flow cytometric data were analyzed with Kaluza software (Beckman Coulter, USA) or FlowJo (FlowJo LLC, Ashland, Oregon, USA).

### Soft-agar colony formation

A soft-agar assay was performed on 12-well plates with a base layer of 0.5% agarose gel containing DMEM. To generate the cell layer, 5 × 10^3^ cells/well were suspended in 0.35% top agarose gel in DMEM/F12 medium containing B27 supplement, 20 ng/ml of EGF and 20 ng/ml of basic FGF. Plates were incubated at 37 °C in 5% CO_2_ for 17 days to allow colony formation, and cell viability was determined by staining with 3-(4,5-dimethylthiazol-2-yl)-2,5-diphenyl tetrazolium bromide (MTT, Sigma-Aldrich, St. Louis, MO, USA, 1 mg/ml). Colonies of each cell line were counted (>100 *μ*m in diameter). All experiments were performed in triplicate, and data are presented as means±S.D.

### Western blot analysis

For western blot analysis, cells were harvested and prepared using RIPA buffer (Sigma-Aldrich) and sub-cellular fractions prepared using the NE-PER Nuclear Protein Extraction Kit (Thermo Scientific, Nepean, ON, Canada) containing protease inhibitor cocktails (Roche, Mannheim, Germany). Protein concentration was determined using a Bio-Rad DC protein assay kit (Bio-Rad, Hercules, CA, USA). Subsequently, 25–30 *μ*g of total protein for each sample was loaded onto an 8-12% SDS-PAGE for electrophoresis and then transferred to a PVDF membrane. Protein was identified by incubating the membrane with primary antibodies, followed by horseradish peroxidase-conjugated secondary antibodies and an enhanced chemiluminescence solution (Pierce, Thermo Scientific, USA). Antibodies used in this study include: anti-c-MYC polyclonal antibody (1:1000, D84C12, Cat. 5605), anti-CD44 (8E2) monoclonal antibody (5640), anti-phospho-Stat3 (Tyr705) monoclonal antibody (1:1000, D3A7, Cat. 9145), anti-Stat3 monoclonal antibody (1:1000,124H6, Cat. 9139)) from Cell Signaling (Danvers, MA, USA); anti-ALDH1A1 antibody (1:1000, ab105920) from ABCAM (Cambridge, UK); anti-*β*-actin monoclonal antibody (1:5000, AC15) from Sigma-Aldrich (St. Louis, MO, USA); anti-Oct4 antibody (ab137427) from ABCAM (Toronto, ON, Canada); Anti-NF-*κ*B p65 monoclonal antibody (1:1000, 112A1021), anti-phospho-NF-*κ*B p65 pSer 536 monoclonal antibody (1:1000, T.849.2), anti-I-kappa-B-alpha monoclonal antibody (1:1000, T.937.7), and anti-phospho-I-kappa-B-alpha pSer32/36 monoclonal antibody (1:1000, H.709.9) from Thermo scientific (Rockford, USA).

### Alamar blue assay

The Alamar Blue assay was used for cell viability or relative cytotoxicity analysis according to manufacturer instructions (Bio-Rad Laboratories GmbH, Munich, Germany). Briefly, Alamar Blue dye (BUF012B, Bio-Rad Laboratories), a cell metabolism indicator, was added to a final concentration of 1% (vol/vol) for 4-6 h. Fluorescence was measured using a Synergy H1 Hybrid Multi-Mode Microplate Reader (BioTek Instruments, Winooski, VT, USA) in top-reading mode with excitation at 560 nm and emission at 590 nm. Readings were analyzed using Omega analysis software. Cells grown in media+vehicle served as a vehicle control whereas media alone containing 1% (vol/vol) Alamar Blue served as blank and subtracted from the fluorescence value obtained for each well. Results were expressed as relative fluorescence (%) after normalized by designating vehicle control (100%) according to the manufacturer's protocol.

### Mammosphere formation assays

SUM190, SUM149 and MDA-MB-231 cells (2 × 10^3^/well) were reseeded in Ultra-Low attachment plates (Costar, Corning, NY, USA) after treated with vehicle-derived or paclitaxel-derived supernatants for 4 days. The 6-well plates were incubated at 37 °C in 5% CO_2_ for 8 days to allow mammosphere formation. Colonies (>100 *μ*m in diameter) of each group were counted.

### Quantitative real-time PCR

Total RNAs were extracted using RNeasy kit (QIAGEN) and real-time qPCR analysis was performed using Bio-Rad MyiQ (Bio-Rad) as previously described.^36, 37^ The conditions for qPCR reactions are: one cycle at 95 °C for 20 s, followed by 40 cycles at 95 °C for 3 seconds and annealing at 60 °C for 30 s. Results were normalized to the housekeeping gene glyceraldehyde 3-phosphate dehydrogenase (*GAPDH*). Relative expression level of genes from different groups were calculated with the ^2ΔΔ^CT method and compared with the expression level of the corresponding gene in control cells, Specific primer sequences for individual genes are listed in Supplementary Table 1.

### Supernatants and human cytokine arrays

Cell-free supernatants were generated from SUM190, SUM149 and MDA-MB-231 cells after 8 days culture. The cells were first cultured in the presence of vehicle or paclitaxel (15 nM), doxorubicin (0.35 *μ*M) or 5-fluorouracil (75 *μ*M) for 4 days. After washed with PBS, the cells were continuously cultured in fresh medium without any drug for additional 4 days. At Day 8, the drug- and cell-free supernatants were collected for subsequent experiments and for human cytokine assay. A 11-plex human cytokine profiling kit was used for human cytokine arrays for protein concentrations of cytokines in the supernatants, including IFN-*γ*, GM-CSF, interleukin 1*β* (IL1*β*), interleukin 2, interleukin 4, interleukin 6, interleukin 8, interleukin10, interleukin 12, CCL2/MCP-1 and tumor necrosis factor *α* (TNF-*α*). The arrays were performed blindly by Eve Technologies (Eve Technologies, Calgary, Alberta, Canada).

### siRNA knockdown

siRNAs for NF-*κ*B p65 and *β*-catenin, and control scrambled siRNA were purchased from Thermo Scientific (Dharmacon, USA) as SMARTpools. For siRNA transfections, cells were transiently transfected with these oligos using Lipofectamine RNAiMAX reagent (Invitrogen) according to the manufacturer’s instructions. Transfection efficiency was assessed using qPCR and western blot. After treatments, cells were trypsinized and subjected to western blot and flow cytometry assays.

### Transfection, transduction and *β*-catenin/TCF-eGFP reporter assays

The Wnt reporter breast cancer subline 7TGP-SUM190 were generated by transduction of Wnt reporter construct 7TGP ^23^ into SUM190 cells by lentiviral transduction. The *β*-catenin/TCF/LEF-dependent reporter plasmid (7 × Tcf-eGFP//SV40-PuroR, 7TGP) containing seven Tcf/Lef-binding sites and a puromycin resistance gene was provided by Dr. Nusse via Addgene (#24305).^23^ Lentiviral production was carried out as described previously.^25, 38^ Briefly, ten 10-cm dishes were seeded with 6 × 10^6^cells per dish overnight before transfection. For two dishes, 8 *μ*g of the lentiviral vector, 5.4 *μ*g of the psPax2 envelope plasmid, 3.6 *μ*g of the packaging plasmid (pMD2.G) were used. The medium was replaced overnight, and lentiviral supernatant was harvested after 48 hours, filtered through a 0.45-*μ*m PES filter, and concentrated with Lenti-X concentrator (Clontech, Mountain View, CA, USA). For viral infection, once SUM190 cells in 6-well plate reached 40-50% confluence, 1 ml of concentrated lentiviral supernatant and 8 *μ*g/ml of polybrene were added for 24 h. The infected cells containing the reporter TCF-eGFP cassette were selected with puromycin. The TCF-eGFP expression levels after treatment with vehicle- or paclitaxel-derived supernatants were determined by FACS.

### Scratch wound-healing assay

Scratch wound-healing assay was performed as reported previously.^27, 39^ Briefly, cells were seeded in 24-well plates and grown to confluence. The monolayer was scratched with a 200 *μ*l sterile pipette tip, washed twice with PBS to remove detached cells and debris, and then incubated with medium containing different reagents as indicated. Each experimental condition was tested in quadruplicate. The cells were photographed at different time points using a Zeiss Axiovert 40 CFL microscope (Carl Zeiss AG, Feldbach, Switzerland). The open area was quantified on each photo to obtain mean values. Migration was reported as the difference (in mm^2^) between the scratch dimensions observed at 0 and 24-h.

### Xenograft tumor growth

All mouse experimentation was conducted in accordance with standard operating procedures approved by the Animal Care Committee at the University of Ottawa. Athymic nude mice (6–8 week old, female, 20 to 25 g body weight) were obtained from Charles River Laboratories. To establish breast cancer xenografts in nude mice, SUM149 cells were mixed with Matrigel (BD Biosciences, Bedford, MA, USA) and injected under aseptic conditions into mammary fat pads of nude mice (*n*=4–8 for each group, 2 × 10^6^ cells per fat pad). The tumor was monitored and evaluated every 2–3 days with calipers. Tumors were measured in 2 dimensions, and volume was calculated according to the formula: *V*=0.5 × (length) × (width)^2^. When the tumor reached a mean diameter of 4 mm (day 15), tumor-bearing mice were randomized into 4 groups and intraperitoneally injected with vehicle, Paclitaxel alone (10 mg/kg on days 15, 20 and 25), reparixin alone (25 mg/kg on every day for 10 days starting from day 25), or paclitaxel following by reparixin for 10 days. At the end of drug treatment, mice were humanely euthanized and tumors were harvested for further analyses and secondary transplantation in a blinded manner.

### Secondary transplantation of nude mouse model

Tumor tissues were dissociated mechanically and enzymatically to obtain a single-cell suspension. Tumors were minced by scalpel and incubated in Hams F12 (Invitrogen) containing collagenase/hyaluronidase (#07912, STEMCELL Technologies, Canada) at 37 °C for 60 min. The tissues were further dissociated by pipette trituration and then passed through a 40-*μ*m nylon mesh to produce a single-cell suspension. Three groups of mice were implanted with tumor cells. Each athymic nude mouse was inoculated into one of the inguinal mammary fat pads with 10^5^, 10^4^, 10^3^ or 10^2^ cells from the first tumors receiving treatment of either vehicle, paclitaxel, or paclitaxel followed by reparixin. Tumor growth and size were measured every other day for 6 weeks.

### Statistical analyses and survival curve

Data are expressed as means±S.D. unless specified elsewhere. Statistical significance was determined using a Student's *t* test, ANOVA or *chi*-square test wherever appropriate. A Kaplan–Meier log-rank test survival plot was performed using the data available at http://www.cbioportal.org/study?id=brca_metabric#summary (Breast Cancer, METABRIC, Nature 2012 & Nature Communications 2016). The log-rank test was performed to determine whether observed differences between groups were statistically significant. Results were considered significant with a *P-*value<0.05.
