# Supplementary material for: An autocrine inflammatory forward-feedback loop after chemotherapy withdrawal facilitates the repopulation of drug-resistant breast cancer cells
Source: Cell Death Dis. 2017 Jul 13;8(7):e2932–. doi: 10.1038/cddis.2017.319 (PMC5550865; doi:10.1038/cddis.2017.319)
Supplement: Supplementary Table 1 [file cddis2017319x1.docx]

**Supplemental Table 1**

**Table S1.** List of primers used in quantitative PCR for measuring gene expression relative to GAPDH (Related to Experimental Procedures)

| **Gene names** | **Forward** | **Reverse** |
| --- | --- | --- |
| Oct4 | CTGCAGTGTGGGTTTCGGGCA | CTTGCTGCAGAAGTGGGTGGAGGAA |
| Nanog | CATGAGTGTGGATCCAGCTTG | CCTGAATAAGCAGATCCATGG |
| IL-6 | AACAACCTGAACCTTCCAAAGA | TCAAACTCCAAAAGACCAGTGA |
| Axin2 | GTCACCAAACCCATGCCTGTCTCT | TAAGCACCGTCTTGATCGCCCAAT |
| Hey1 | CGAGGTGGAGAAGGAGAGTG | TCGGCGCTTCTCAATTATTC |
| IKBa | CTCCGAGACTTTCGAGGAAATAC | GCCATTGTAGTTGGTAGCCTTCA |
| IL-8 | ATGACTTCCAAGCTGGCCGTGGCT | TCTCAGCCCTCTTCAAAAACTTCTC |
| Klf4 | CAAGTCCCGCCGCTCCATTACCAA | CCACAGCCGTCCCAGTCACAGTGG |
| CCL-2 | AAGATCTCAGTGCAGAGGCTCG | TTGCTTGTCCAGGTGGTCCAT |
| MMP9 | TTGACAGCGACAAGAAGTG | CTGAGGAATGATCTAAGCC |
| CSF2 | ATGATGGCCAGCCACTACAA | CTGGCTCCCAGCAGTCAAAG |
| ALDH1 | CGCAAGACAGGCTTTTCAG | TGTATAATAGTCGCCCCCTCTC |
| CD44 | AGACAACCACAAGGATGACTGATG | TCCAGTTTCCTTCATAAGCAGTGG |
| c-Myc | TTCTCTCCGTCCTCGGATTCTCTG | TCTTCTTGTTCCTCCTCAGAGTCG |
| Sox2 | CATCACCCACAGCAAATGACAGC | TTGCGTGAGTGTGGATGGGATTG |
| Nanog | CATGAGTGTGGATCCAGCTTG | CCTGAATAAGCAGATCCATGG |
| Slug | TGTTGCAGTGAGGGCAAGAA | GACCCTGGTTGCTTCAAGGA |
| Snail | TGCAGGACTCTAATCCAAGTTTACC | GTGGGATGGCTGCCAGC |
| ZEB1 | GCCAATAAGCAAACGATTCTG | TTTGGCTGGATCACTTTCAAG |
| E-cadherin | TGCCCAGAAAATGAAAAAGG | GGATGACACAGCGTGAGAGA |
| ABCB1 | GCCTGGCAGCTGGAAGACAAATAC | ATGGCCAAAATCACAAGGGTTAGC |
| GAPDH | ACAGTCAGCCGCATCTTCTT | GACAAGCTTCCCGTTCTCAG |
