# Supplementary material for: An autocrine inflammatory forward-feedback loop after chemotherapy withdrawal facilitates the repopulation of drug-resistant breast cancer cells
Source: Cell Death Dis. 2017 Jul 13;8(7):e2932–. doi: 10.1038/cddis.2017.319 (PMC5550865; doi:10.1038/cddis.2017.319)
Supplement: Supplementary Figures [file cddis2017319x2.pdf]

Figure S1

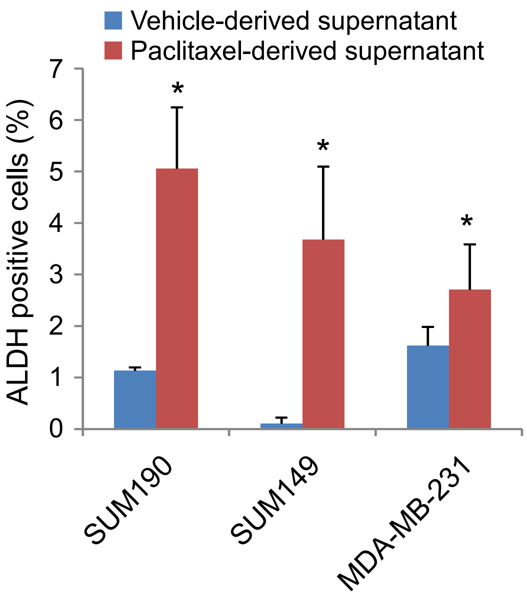

Figure S1. Paclitaxel-derived supernatants enhance stem-like cell phenotypes (related to Figure 1). Flow cytometric analysis of the percentage of ALDH-positive cells in SUM190, SUM149 and MDA-MB-231 breast cancer cells after exposure to vehicle-derived or paclitaxel-derived supernatants for 4 days. Pre-gated on 7-AAD-negative live cells. Data represent means ± SD, n = 3; * $p < 0.05$.

Figure S2

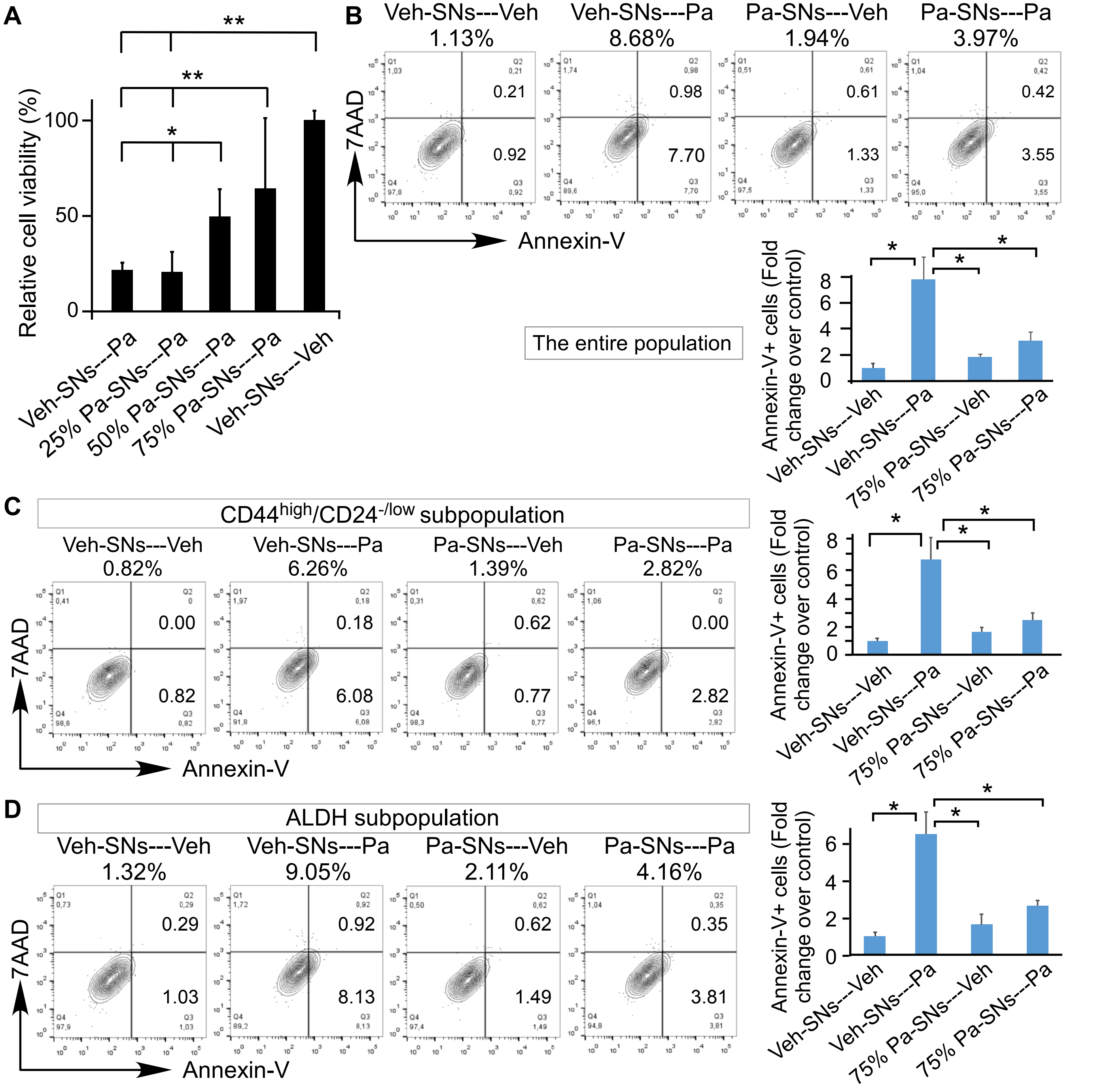

Figure S2. Breast cancer cells become more resistant to paclitaxel treatment (related to Figure 1). (**A**) SUM149 cells were cultured in the medium containing different proportions of vehicle-derived supernatants (Veh-SNs) or paclitaxel-derived supernatants (Pa-SNs) for 4 days and then treated with vehicle (Veh) or 5 nM paclitaxel (Pa) for 3 days (7 days in total). Relative cell viability was assessed by Alamar blue assay at day 7 by comparison to control group (Veh-SNs---Veh, 100% viability at day 7). (**B-D**) Flow cytometric analysis of apoptotic cells on the entire population (B), CD44^{high}/CD24$^{-/low}$ subpopulation (C), or ALDH+ subpopulation (D) at day 5 after culture in the medium containing 75% of Veh-SNs or Pa-SNs for 4 days followed by treatment with Veh or 5 nM paclitaxel for 1 day (5 days in total). Cells were stained with Annexin-V and 7AAD. Cells were pre-gated on CD44^{high}/CD24$^{-/low}$ or ALDH+ subpopulation for C or D. The contour/dot plots are all from one experiment and histograms from three independent experiments showing fold change of apoptotic cells (including early and late apoptotic cells) over the control (Veh-SNs---Veh) group. Data are means ± SD, n = 3; * $p < 0.05$.

Figure S3

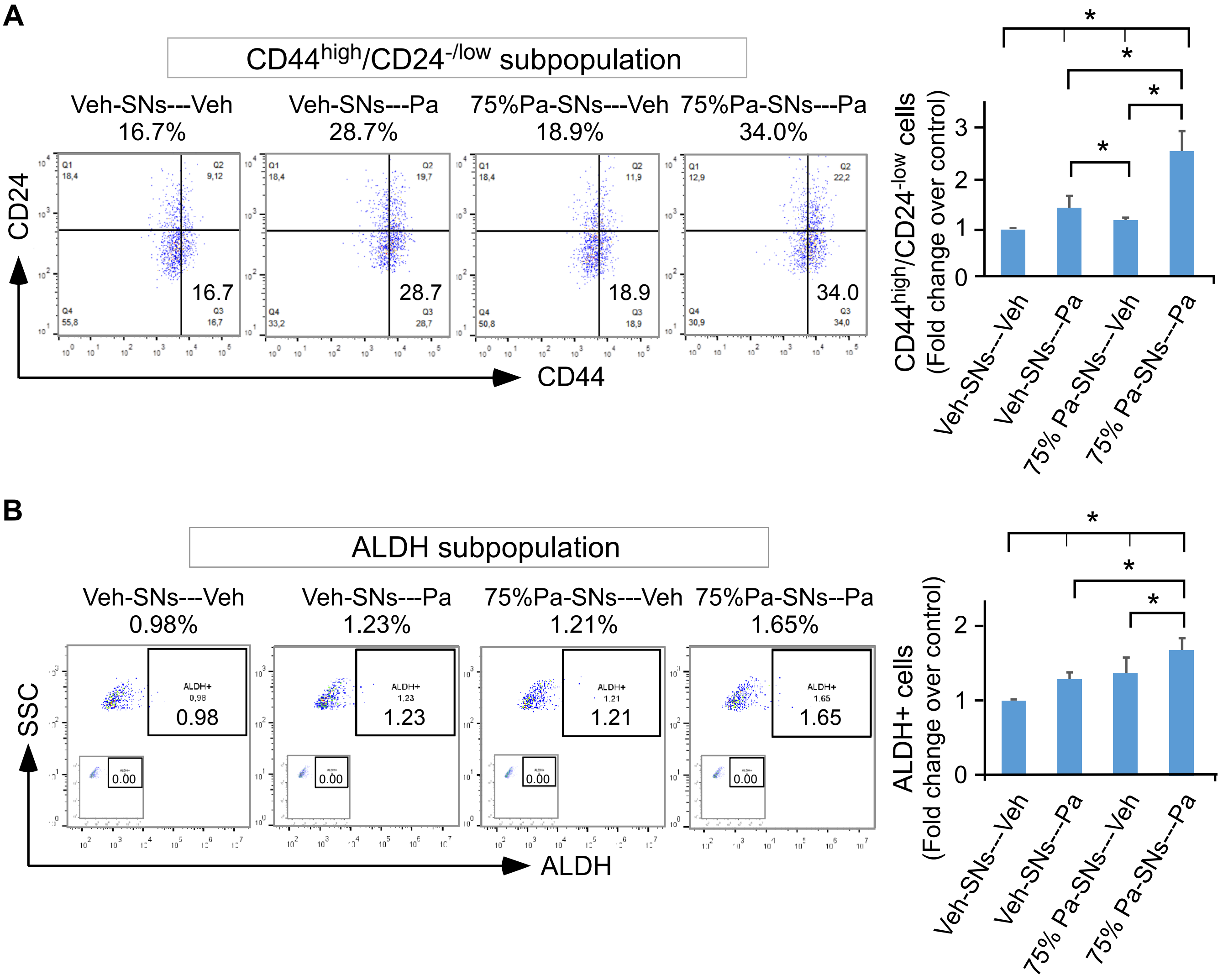

Figure S3. Breast cancer cells become more resistant to paclitaxel treatment and enrich more CSCs after pre-exposure to paclitaxel-derived supernatants (related to Figure 1). SUM149 cells were cultured in the medium containing 75% of vehicle-derived supernatants (Veh-SNs) or paclitaxel-derived supernatants (Pa-SNs) for 4 days and then treated with vehicle (Veh) or 5 nM paclitaxel (Pa) for 3 days. $CD44^{high}/CD24^{-/low}$ and ALDH+ subpopulations were analyzed at day 7 by flow cytometry after different treatments. The 7AAD-negative live cells were pre-gated. The dot plots are from one representative experiment in which cells were stained either for CD44 and CD24, or ALDH markers. The insets in B show the background values of ALDH in each group. Histograms represent means ± SD of at least three independent experiments; * $p < 0.05$, ** $p < 0.01$.

Figure S4

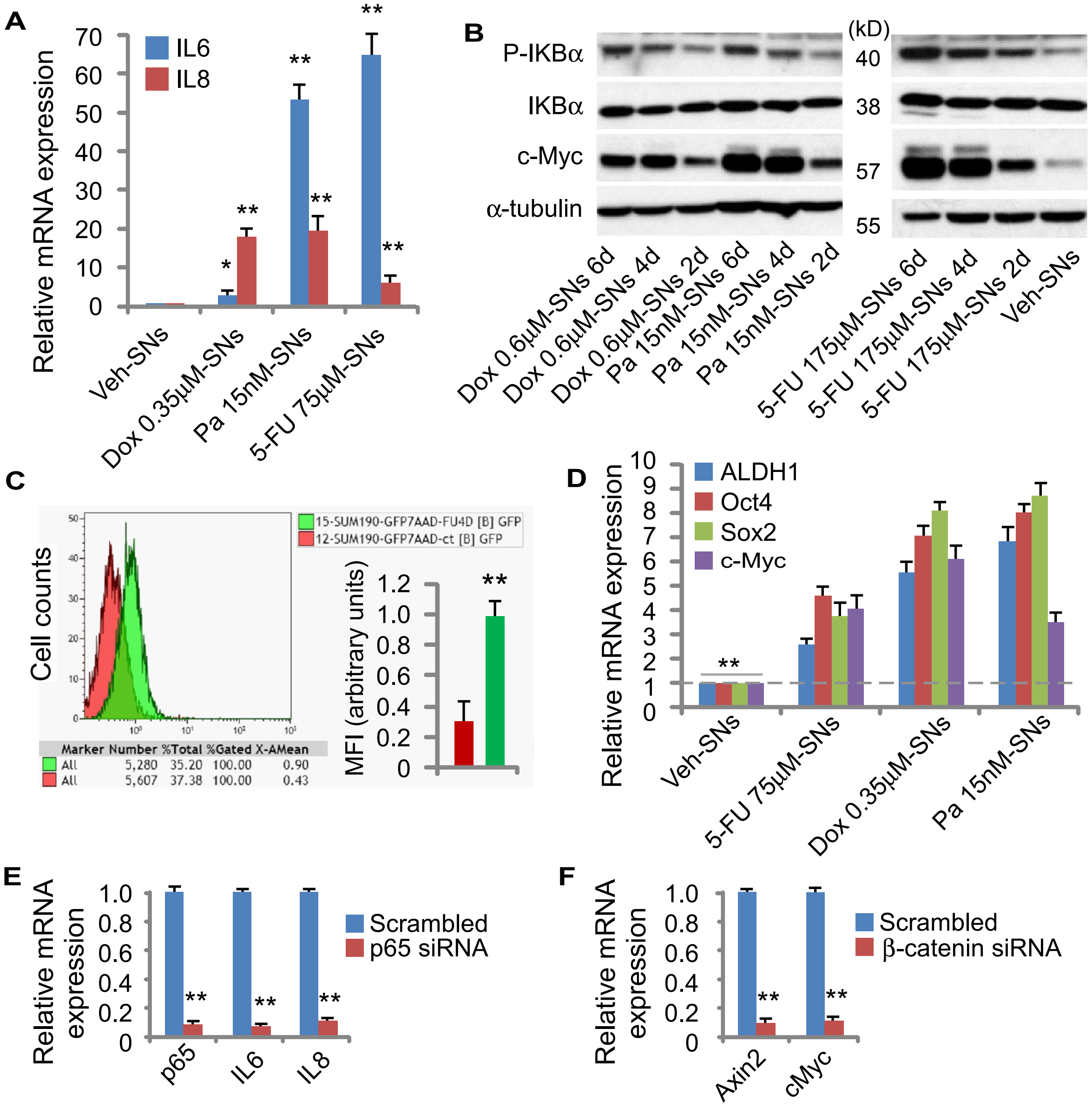

Figure S4. An autocrine inflammatory forward-feedback loop is also observed when cells are exposed to doxorubicin and 5-fluorouracil (related to Figure 3). (**A**) Similar to the cells cultured in paclitaxel-derived supernatants (Pa 15nM-SNs), SUM190 cells cultured in doxorubicin-derived supernatants (Dox 0.35µM-SNs) or 5-fluorouracil-derived supernatants (5-FU 75µM-SNs) were also capable of upregulating the gene expression of *IL8* and *IL6*. (**B**) Doxorubicin-derived and 5-fluorouracil-derived supernatants also enhanced IKBα phosphorylation (P-IKBα, indicating activation of NF-kB pathway), and upregulated the expression of stem cell-associated cMyc protein. (**C**) 5-fluorouracil-derived supernatant also upregulated Wnt signaling pathway. Flow cytometric analysis of 7xTCF-eGFP reporter activity in Wnt reporter subline 7xTCF-SUM190 in the presence of vehicle-derived supernatants (12-SUM190-GFP7AAD-ct, red)-derived or 5-fluorouracil-derived supernatants for 4 days (15-SUM190-GFP7AAD-FU4D, green). Live cells were pre-gated on 7-AAD negative cells (representing live cells). TCF-eGFP reporter activity was measured by the percentage of GFP positive cells and mean fluorescence intensity (MFI) after different treatments. (**D**) Similar to paclitaxel-derived supernatants, doxorubicin-derived and 5-fluorouracil-derived supernatants enhanced the expression of CSC-associated genes. (**E** and **F**) Knockdown efficiency of NF-kB (p65 siRNA) and β-catenin (β-catenin siRNA) in SUM149 cells was confirmed by significant downregulation of target genes. Data represent means ± SD, n = 3; * $p < 0.05$, ** $p < 0.01$ between control and treated groups.

Figure S5

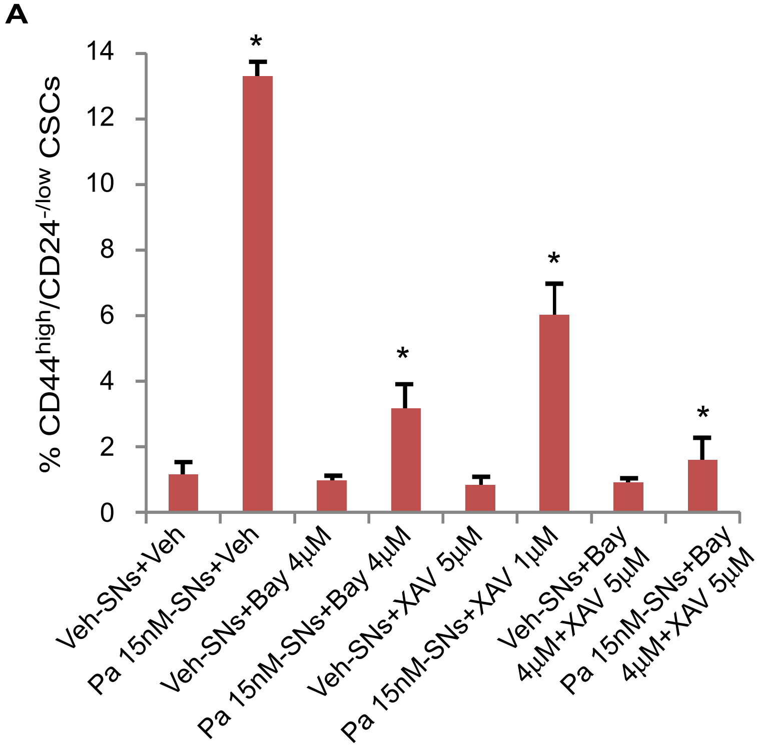

Figure S5A. NF-kB inhibitor Bay-11-7821 or Wnt/β-catenin inhibitor XAV939 suppresses CD44^{high}/CD24$^{-/low}$ CSC subpopulation induced by paclitaxel-derived supernatants (related to Figure 3). SUM149 cells were pretreated for 2 hours with vehicle (Veh), NF-kB inhibitor Bay-11-7821 (Bay 4μM) and/or Wnt/β-catenin inhibitor XAV939 (XAV 5μM), followed by exposure to vehicle-derived (Veh-SNs) or paclitaxel-derived supernatants (Pa 15nM-SNs) for 4 days in the absence or presence of the same vehicle or inhibitors. At day 4, cells were stained with fluorophore-cojugated CD44 and CD24 antibodies and 7-AAD (excluding dead cells) and analyzed by flow cytometry. Data are means ± SD, n = 3; $p < 0.05$ in comparison to each control group.

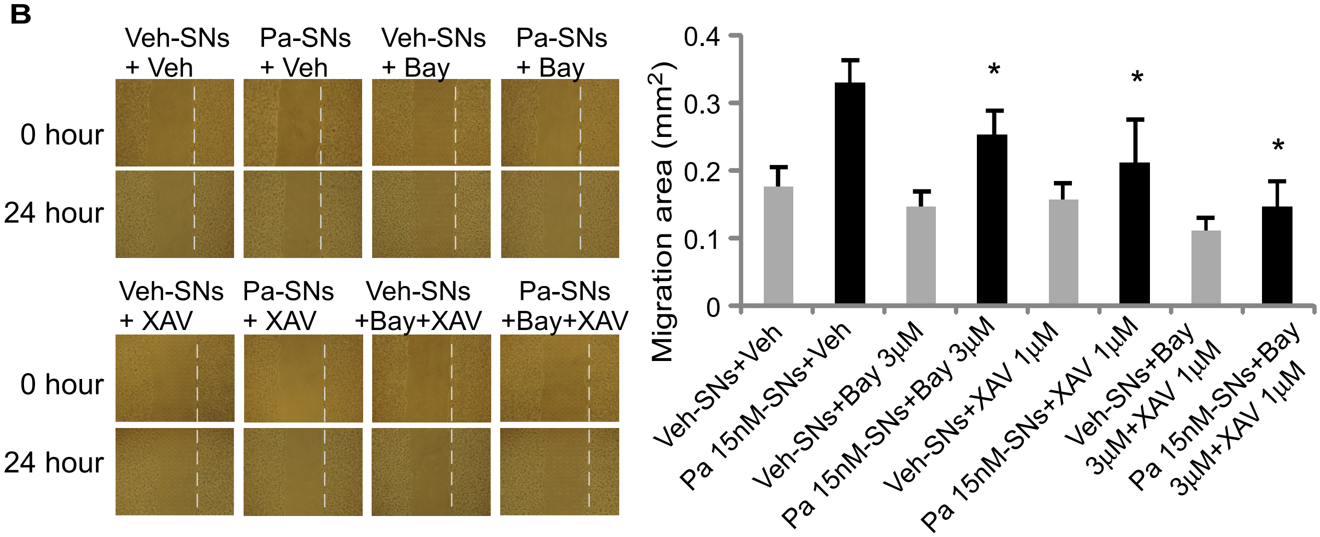

Figure S5B. NF-kB inhibitor Bay-11-7821 and/or Wnt/β-catenin inhibitor XAV939 suppresses migration of breast cancer cells induced by paclitaxel-derived supernatants (wound-healing assays, related to Figure 3). SUM149 cells were grown to confluence, pretreated with vehicle (Veh), XAV939 (XAV) and/or Bay-11-7821(Bay) for 2 hours, scratched, and then observed for wound healing migration after exposure to vehicle (Veh)- or paclitaxel(Pa)-derived supernatants (SNs) in the presence of the same vehicle or inhibitors. Representative scratch healing images are shown. Histogram represents migration (in mm^2) at 24-hour after scratch. Dashed lines indicate wound edges on the right. Data represent means ± SD, n = 4; *$p < 0.05$ in comparison to Pa 15nM-SNs+Veh group.
